# Theoretical Investigation of the Formation Mechanism of NH_3_ and HCN during Pyrrole Pyrolysis: The Effect of H_2_O

**DOI:** 10.3390/molecules23040711

**Published:** 2018-03-21

**Authors:** Ji Liu, Qiang Lu, Xiao-yan Jiang, Bin Hu, Xiao-lei Zhang, Chang-qing Dong, Yong-ping Yang

**Affiliations:** 1 National Engineering Laboratory for Biomass Power Generation Equipment, North China Electric Power University, Beijing 102206, China; liujipower@126.com (J.L.); jiangxy90@126.com (X.-y.J.); binhu92@126.com (B.H.); cqdong1@163.com (C.-q.D.); yyp@ncepu.edu.cn (Y.-p.Y.); 2 School of Mechanical and Aerospace Engineering, Queen’s University of Belfast, Belfast BT9 5AH, UK

**Keywords:** pyrrole pyrolysis, H_2_O, NO_x_ precursor, NH_3_, HCN, DFT

## Abstract

Coal is a major contributor to the global emission of nitrogen oxides (NO_x_). The NO_x_ formation during coal utilization typically derives from the thermal decomposition of N-containing compounds (e.g., pyrrolic groups). NH_3_ and HCN are common precursors of NO_x_ from the decomposition of N-containing compounds. The existence of H_2_O has significant influences on the pyrrole decomposition and NO_x_ formation. In this study, the effects of H_2_O on pyrrole pyrolysis to form NO_x_ precursors HCN and NH_3_ are investigated using the density functional theory (DFT) method. The calculation results indicate that the presence of H_2_O can lead to the formation of both NH_3_ and HCN during pyrrole pyrolysis, while only HCN is formed in the absence of H_2_O. The initial interaction between pyrrole and H_2_O determines the N products. NH_3_ will be formed when H_2_O attacks the C_2_ position of pyrrole with its hydroxyl group. On the contrary, HCN will be generated instead of NH_3_ when H_2_O attacks the C_3_ position of pyrrole with its hydroxyl group. In addition, the DFT calculations clearly indicate that the formation of NH_3_ will be promoted by H_2_O, whereas the formation of HCN is inhibited.

## 1. Introduction

Coal is the major energy source in the modern industrial production process. Coal-mining, coal-burning factories, and other coal-related usage are the dominant sources of NO_x_ emission [1,2]. The harmful atmospheric pollutants of NO_x_ (i.e., NO, NO_2_, N_2_O, etc.) can form acid rain and photochemical smog and endanger human health [3,4,5]. Moreover, the contribution of NO to the greenhouse effect is 260 times higher than that of CO_2_ [6].

Nitrogen compounds in coal are primarily pyrrole and pyridine, which have already been widely used in different coal structural models, such as the Given, Wiser, and Shinn models [7,8,9]. The well-known Given model, mainly containing carbon, hydrogen, oxygen, and small amounts of sulfur and nitrogen, was intended to show the types of hydrogen structures in the bituminous coal, and has been widely adopted as a structural representation. The Wiser mode,l along with “structural alternative” carbon skeletal representations, had an increasing scale, representing the rank transition from 76% C to 90% C (wt % basis) [9]. The Shinn model was created at a larger scale (10,000 amu), in which three relatively small unconnected molecular entities were held within a larger molecule [8]. It is notable that around 60% of total coal nitrogen is the pyrrolic nitrogen [10], and this concentration varies slightly with different coal ranks [10,11]. Hence, the thermal decomposition of pyrrolic groups plays a vital role in NO_x_ formation. As a result, pyrrole was commonly employed as the N-containing model compound in coal, and has been extensively studied in recent decades.

Many efforts have been made to characterize the pyrolysis of pyrrole using experimental and theoretical approaches [12,13,14,15,16,17]. Martoprawiro et al. [12] proposed a mechanism for the formation of HCN from pyrrole, which stated that the reaction was initiated by a hydrogen migration to form a cyclic carbene intermediate. Subsequent ring opening and decomposition steps gave rise to HCN and propyne. Moreover, Zhai et al. [18] investigated the mechanisms of pyrrole pyrolysis, outlining the routes for formation of cis-crotonitrile, allyl cyanide, and HCN. Besides HCN, NH_3_ is another important precursor of NO_x_ from coal utilization at high temperatures [19,20,21]. Li et al. [20] conducted pyrolysis experiments on coal and concluded that the formation of both HCN and NH_3_ was initiated by the N-containing heteroaromatic ring being attacked by radicals. Similarly, Elmer et al. [21] employed a quartz fluidized-bed reactor coupled to a quartz tubular flow reactor for rapid pyrolysis of coal, and suggested that NH_3_ might be formed from the interactions of N-containing species with donatable H on the soot surface. However, to date, few theoretical studies have focused on the formation of NH_3_ during pyrrole pyrolysis.

During the coal pyrolysis process, H_2_O will play an essential role in the decomposition reactions and formation of various products. Hu et al. [22] investigated the pyrolysis behavior and reaction mechanisms of coal samples containing different moisture contents. They found that the moisture content had a significant influence on the tar yield and light tar content, and H_2_O could upgrade the low-rank coal during pyrolysis [23]. Moreover, Park and co-workers [24] found that studies of coal-N release in the presence of H_2_O were more authentic than those of the only pyrrole, as H_2_O inevitably existed in the coal utilization process. However, there are limited studies focusing on the effect of H_2_O on pyrrole pyrolysis at present. Therefore, in this study, the density functional theory (DFT) method was applied to investigate the detailed pyrolysis mechanism of pyrrole to HCN and NH_3 _in the presence of H_2_O, which may help with the development of coal pyrolysis/combustion technologies for NO_x_ control.

## 2. Results

**Figure 1:**
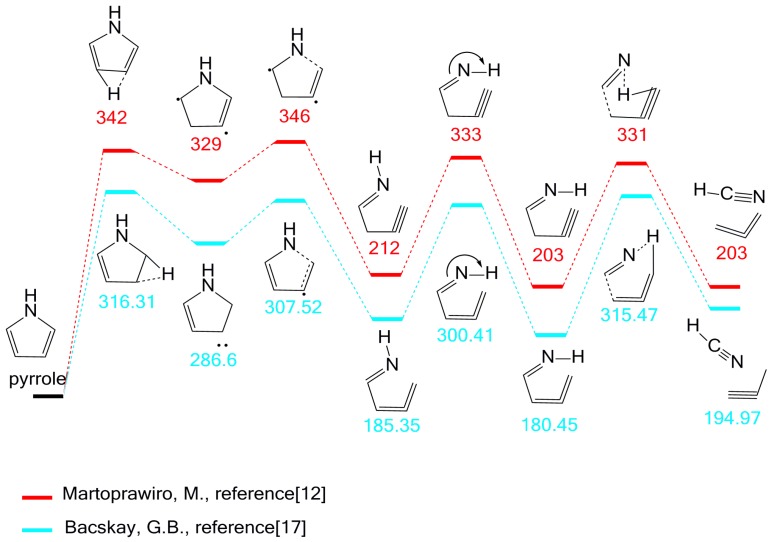
The proposed pyrolysis mechanism of pure pyrrole in previous studies. The exact digits follow the original paper. The numerical values are energies in kJ/mol.

Early studies [12,17,25] have already investigated the possible pyrolysis pathways of pure pyrrole using quantum chemistry methods. The obtained activation energies (346 and 316.31 kJ/mol, as shown in Figure 1) are comparable with the activation energy measured from the experimental energy of the overall disappearance of pyrrole between 1300 and 1500 K at 12 atm pressure (310.1 ± 12.5 kJ/mol) [19]. The activation energy difference between the two pathways in Figure 1 is mainly due to the initial H migration to different carbons. In the proposed pathways, the crucial step is the isomerization of pyrrole to the cyclic carbine. Then, the primary N-type product of HCN is formed through a ring opening reaction, as shown in Figure 1. No NH_3_ will be formed in this pyrolysis process.

**Figure 2:**
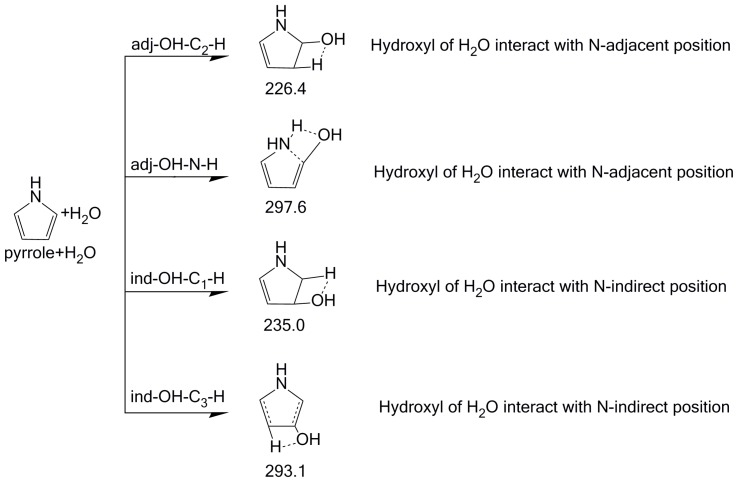
Initial interaction and pyrolysis mechanism of pyrrole in the presence of H_2_O.

When H_2_O participates in the pyrrole pyrolysis process, it will interact with pyrrole, thus significantly altering the pyrolysis mechanism and pathways. As shown in Figure 2, the possible interactions between pyrrole and H_2_O may occur in four ways, i.e., adj-OH-C_2_-H, adj-OH-N-H, ind-OH-C_1_-H, and ind-OH-C_3_-H (the adjacent position is abbreviated as adj-, the indirect position is abbreviated as ind-), in which H_2_O attacks different positions of pyrrole. In the adj-OH-C_2_-H interaction, H_2_O reacts with pyrrole by simultaneously donating its hydroxyl group to the C_1_ position and hydrogen to the C_2_ position. The energy barrier of this reaction is 226.4 kJ/mol. In an alternate possible interaction, adj-OH-N-H, H_2_O also donates its hydroxyl group to the C_1_ position of pyrrole. Differently, H_2_O provides its hydrogen to the N position, which will result in the opening of the ring. This reaction needs to overcome a higher energy barrier of 297.6 kJ/mol compared to the first reaction (interaction adj-OH-C_2_-H). In the possible ind-OH-C_1_-H interaction, H_2_O also interacts with pyrrole at the C_1_ and C_2_ position. Different from the first reaction, the hydroxyl group attacks the C_2_ position, whereas hydrogen attacks the C_1_ position in this reaction. The energy barrier is 235.0 kJ/mol, which is close to that of the first reaction. In the possible ind-OH-C_3_-H interaction, H_2_O reacts with pyrrole at the C_2_ and C_3_ position with an energy barrier of 293.1 kJ/mol. In summary, based on Figure 2, the initial interaction modes of adj-OH-C_2_-H and ind-OH-C_1_-H are more feasible than the other two interaction modes because of the relatively low energy barriers (226.4 and 235.0 kJ/mol). The possible subsequent pyrolysis pathways based on the four initial interaction ways are presented below to investigate the formation of NH_3_ or HCN.

### 2.1. The Effect of H_2_O on Pyrrole Pyrolysis Based on the adj-OH-C_2_-H Interaction

**Figure 3:**
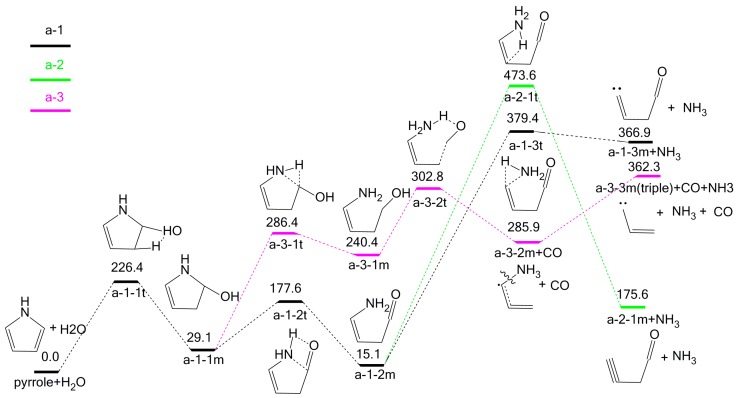
Pyrolytic reaction pathways based on the adj-OH-C_2_-H interaction to generate NH_3_.

**Figure 4:**
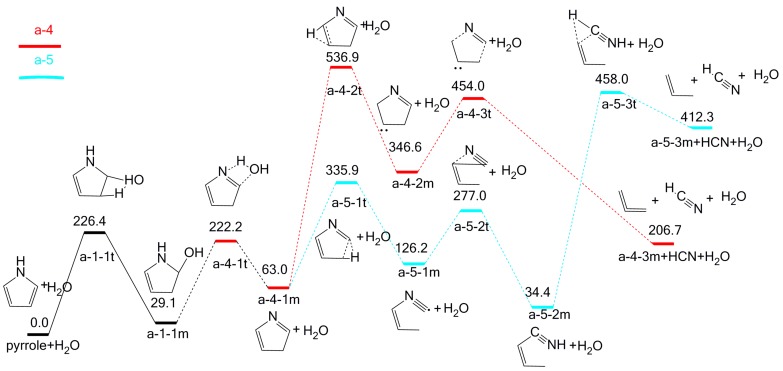
Pyrolytic reaction pathways based on adj-OH-C_2_-H interaction to generate HCN.

Based on the adj-OH-C_2_-H interaction, five possible pathways might occur and can be classified into two categories according to their products (NH_3_ or HCN). NH_3_ is the product in three pathways (a-1, a-2, and a-3), as shown in Figure 3, while HCN is the product in the other two pathways (a-4, a-5), as shown in Figure 4.

In Figure 3, the concerted interaction adj-OH-C_2_-H generates a-1-1m, which may undergo three possible subsequent pathways to form NH_3_. In pathway a-1, intermediate a-1-2t is converted to intermediate a-1-2m, via concerted ring-opening transition state a-1-2t, with an energy barrier of 148.5 kJ/mol. Then intermediate a-1-2m undergoes a hydrogen transfer reaction via three-membered transition state a-1-3t to form products NH_3_ and a-1-3m, with an energy barrier of 364.3 kJ/mol. In pathway a-2, a-1-2m undergoes another hydrogen transfer reaction via four-membered transition state a-2-1t, transferring H from C_3_ position to N position, breaking the C–N bond to form products a-2-1m and NH_3_ with an energy barrier of 458.5 kJ/mol. In pathway a-3, a-1-1m is transformed into intermediate a-3-1m, with an energy barrier of 257.3 kJ/mol. Afterwards, intermediate a-3-1m removes the carbon monoxide through transition state a-3-2t to form the intermediate a-3-2m, with an energy barrier of 62.4 kJ/mol. Finally, a-3-2m decomposes into a-3-3m and NH_3_ by homolysis of the C–N bond, with an energy barrier of 76.4 kJ/mol. Based on the above calculation results, pathway a-3 is the most favorable one due to having the lowest overall energy barrier (362.3 kJ/mol). It is notable that previous studies have indicated that only HCN would be formed from pyrolysis of pyrrole or pyroxene-type products in the absence of H_2_O [12,17,18], whereas the participation of H_2_O in pyrrole pyrolysis can provide hydrogen sources leading to the formation of NH_3_, which is consistent with previous experimental results [24].

Based on the adj-OH-C_2_-H interaction, HCN is the only N product formed through the possible pathways a-4 and a-5. As shown in Figure 4, intermediate a-1-1m undergoes a dehydration reaction between the H proton at N position and the hydroxyl group through transition state a-4-1t. This reaction results in the formation of intermediate a-4-1m, overcoming an energy barrier of 193.1 kJ/mol. For a-4-1m, it has two possible cracking pathways (a-4, a-5). In pathway a-4, intermediate a-4-1m undergoes hydrogen transfer reaction through transition state a-4-2t to form intermediate a-4-2m, with an energy barrier of 474.0 kJ/mol. Then intermediate a-4-2m decomposes into HCN and the a-4-3m through transition state a-4-3t with an energy barrier of 107.4 kJ/mol. The rate-determining step (the reaction bearing the highest energy barrier among all the reactions in an individual pathway) is the reaction of a-4-2m formation, and the overall energy barrier of pathway a-4 is 536.0 kJ/mol. In pathway a-5, intermediate a-4-1m is converted into intermediate a-5-1m via a ring-opening transition state a-5-1t, overcoming an energy barrier of 272.9 kJ/mol. Then, intermediate a-5-1m undergoes further C-N shift reaction through transition state a-5-2t to form intermediate a-5-2m with an energy barrier of 150.8 kJ/mol. Finally, intermediate a-5-2m decomposes into HCN and a-5-3m through transition state a-5-3t with an energy barrier of 423.6 kJ/mol. The rate-determining step is the reaction to generate HCN (the reaction with the highest energy), and the overall energy barrier of pathway a-5 is 458.0 kJ/mol. It is clear that pathway a-5 is more feasible than pathway a-4 by comparing their whole energy barriers.

According to the above calculation results, H_2_O has a significant effect on the pyrolysis of pyrrole. It results in the formation of NH_3_ as a new product, which will not be formed in the absence of H_2_O [12,18,23]. Moreover, H_2_O can also affect the HCN formation. The energy barrier for the generation of HCN in the presence of H_2_O is higher than in the absence of H_2_O [12,17] (details in Figure 1), which indicates that H_2_O can inhibit the formation of HCN during the pyrrole pyrolysis process.

### 2.2. The Effect of H_2_O on Pyrrole Pyrolysis Based on the adj-OH-N-H Interaction

**Figure 5:**
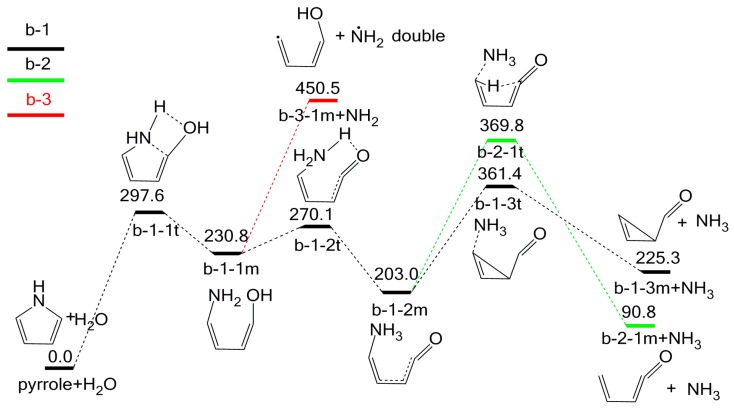
Pyrolytic reaction pathways based on the adj-OH-N-H interaction.

Following the initial adj-OH-N-H interaction, three possible routes might occur, i.e., b-1, b-2, and b-3, as shown in Figure 5. All three pathways result in the formation of NH_3_ rather than HCN.

As stated above, the model compound pyrrole and H_2_O pass through transition state b-1-1t with an energy barrier of 297.6 kJ/mol to generate intermediate b-1-1m. Then intermediate b-1-2m is formed with an energy barrier of 39.3 kJ/mol. In this reaction, the hydroxyl H migrates to N position via the four-membered transition state b-1-2t. Following intermediate b-1-2m, there are two possible pathways to produce NH_3_. One is to generate b-1-3m and NH_3_ via transition state b-1-3t, with an energy barrier of 158.4 kJ/mol (pathway b-1). The other is to form b-2-1m and NH_3_ through transition state b-2-1t, with an energy barrier of 166.8 kJ/mol (pathway b-2). In pathway b-3, intermediate b-1-1m decomposes into NH_2_ radical and b-3-1m through homolysis of C-N bond, with an energy barrier of 219.7 kJ/mol. NH_2_ radical can easily form NH_3_ by capturing the H radical in the pyrolysis process. Among the three pathways, pathway b-1 is the most feasible one, with the overall energy barrier of 361.4 kJ/mol (369.8 and 450.5 kJ/mol for pathways b-2 and b-3, respectively). From Figure 3 and Figure 5, it can be seen that NH_3_ can be produced in different pathways in the presence of H_2_O.

### 2.3. The Effect of H_2_O on Pyrrole Pyrolysis Based on the ind-OH-C_1_-H Interaction

**Figure 6:**
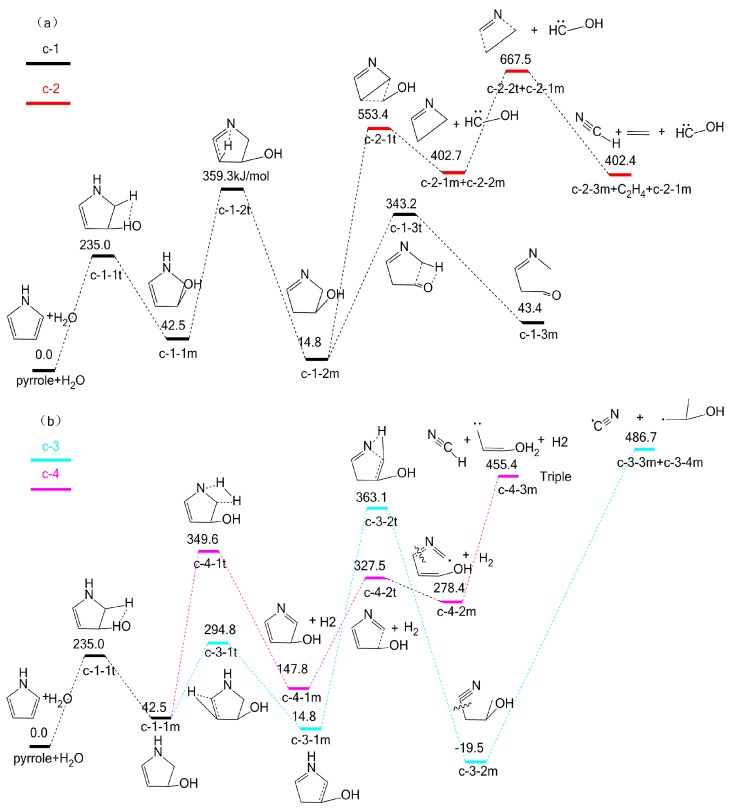
Pyrolytic reaction pathways based on the ind-OH-C_1_-H interaction.

Following the initial ind-OH-C_1_-H interaction, four possible pyrolytic pathways, i.e., c-1, c-2, c-3 and, c-4 might take place, as shown in Figure 6. In Figure 6a, pyrrole and H_2_O interact to generate intermediate c-1-1m, through transition state c-1-1t, with an energy barrier of 235.0 kJ/mol. Then intermediate c-1-1m undergoes a hydrogen transfer reaction via transition state c-1-2t in which the H at the N position migrates to the C_3_ position. This reaction results in the formation of c-1-2m with an energy barrier of 316.8 kJ/mol. Apparently, intermediate c-1-2m will decompose through two possible pathways (pathways c-1 and c-2). In pathway c-1, intermediate c-1-2m forms c-1-3m by migrating hydroxyl H, with an energy barrier of 328.4 kJ/mol. However, possible subsequent pathways of c-1-3m into HCN or NH_3_ cannot be found. In pathway c-2, intermediate c-1-2m is converted into intermediate c-2-1m and c-2-2m via four-membered ring transition state c-2-1t, with an energy barrier of 538.6 kJ/mol. Then intermediate c-2-2m undergoes further reaction via transition state c-2-2t, with an energy barrier of 264.8 kJ/mol, finally decomposing into products HCN, c-2-1m, and c-2-3m.

As shown in Figure 6b, there are two possible subsequent cracking pathways (pathways c-3 and c-4) following intermediate c-1-1m. In pathway c-3, intermediate c-1-1m is converted into intermediate c-3-1m, via transition state c-3-1t, with an energy barrier of 252.3 kJ/mol. Then intermediate c-3-1m undergoes ring-opening reaction via transition state c-3-2t to form intermediate c-3-2m, with an energy barrier of 348.3 kJ/mol. Intermediate c-3-2m finally decomposes into c-3-3m (CN radical) and c-3-4m, with an energy barrier of 486.7 kJ/mol. Then the CN radical will form HCN easily by capturing the H radical. In pathway c-4, intermediate c-1-1m undergoes dehydrogenation via transition state c-4-1t in which H fission from the C_1_ position and N position forms H_2_ and c-4-1m, with an energy barrier of 307.1 kJ/mol. Then intermediate c-4-1m generates intermediate c-4-2m via a five-membered ring-opening transition state c-4-2t, with an energy barrier of 179.7 kJ/mol. Intermediate c-4-2m finally decomposes into c-4-3m and HCN, with an energy barrier of 177.0 kJ/mol. The activation energy of the rate-determining step in pathway c-4 is 455.4 kJ/mol, which is the lowest among the four pathways. It should be pointed out that the activation energy (455.4 kJ/mol) is higher than that of the pyrolysis of hemicellulose, cellulose, or lignin [26]. The main reason can be attributed to the different initial reaction model compounds. The pyrrole structure is highly stable with a five-membered ring, while compounds like hemicellulose and cellulose contain active elements such as oxygen and hydroxyl groups that have promoting effects on the pyrolysis reactions [27]. In summary, the ind-OH-C_1_-H interaction inhibits the formation of HCN, but does not lead to the formation of NH_3_.

### 2.4. The Effect of H_2_O on Pyrrole Pyrolysis Based on the ind-OH-C_3_-H Interaction

**Figure 7:**
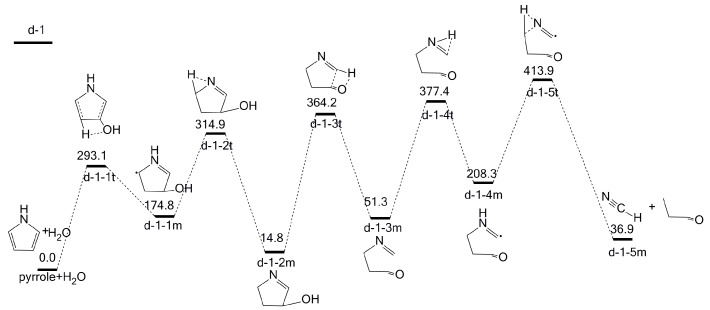
Pyrolytic reaction pathways based on the ind-OH-C_3_-H interaction.

As shown in Figure 7, following the ind-OH-C_3_-H interaction, there is only one possible pathway, d-1, which results in the formation of only HCN.

The initial ind-OH-C_3_-H interaction leads to the formation of intermediate d-1-1m through transition state d-1-1t, with an energy barrier of 293.1 kJ/mol. Then, d-1-1m forms intermediate d-1-2m via transition state d-1-2t with an energy barrier of 140.1 kJ/mol. In this reaction, the hydrogen at N position migrates to the adjacent C_4_ position. Intermediate d-1-2m further generates intermediate d-1-3m through transition state d-1-3t, with an energy barrier of 349.4 kJ/mol. Afterwards, intermediate d-1-3m undergoes hydrogen transfer reaction via transition state d-1-4t to form intermediate d-1-4m, with an energy barrier of 326.1 kJ/mol. Finally, intermediate d-1-4m decomposes into HCN and d-1-5m, with an energy barrier of 205.6 kJ/mol. The rate-determining step is the last step, and the overall energy barrier of pathway d-1 is 413.9 kJ/mol. Notably, both pathways following interactions ind-OH-C_1_-H and ind-OH-C_3_-H do not lead to the formation of NH_3_, implying that NH_3_ cannot be formed when the hydroxyl group of H_2_O attacks the C_3_ position.

## 3. Discussion

The above calculation clearly indicates that H_2_O plays a vital role in the pyrolysis of pyrrole. The type of N products will be determined by the initial interaction of H_2_O and pyrrole. Based on the calculation results, the optional pathways following each initial interaction of H_2_O and pyrrole are summarized in Table 1. Following the initial adj-OH-C_2_-H interaction, the optimal pathway is a-3, with an overall energy barrier of 362.3 kJ/mol, and the N product is NH_3_. Within the initial adj-OH-N-H interaction, pathways a-3 and b-1 are the optimal pathways for the formation of NH_3_. The overall energy barriers are 362.2 and 361.4 kJ/mol, respectively. Starting from the initial ind-OH-C_1_-H interaction, the optimal pathway is c-4, with an overall energy barrier of 455.4 kJ/mol, and HCN is the final product. Following the initial ind-OH-C_3_-H interaction, there is only one possible pathway (pathways d-1) for HCN formation, with an overall energy barrier of 413.9 kJ/mol. Compared with the HCN formation from pure pyrrole pyrolysis process (shown in Figure 1), generation of HCN in the presence of H_2_O needs to overcome a higher energy barrier [12,17]. Hence, the presence of H_2_O in pyrrole pyrolysis inhibits the formation of HCN, while promotes the formation of NH_3_. For the two N products of NH_3_ and HCN, the formation pathways of NH_3_ need to overcome lower overall energy barriers than those of HCN. Therefore, it can be concluded that formation of NH_3_ is easier than that of HCN during the pyrrole pyrolysis process in the presence of H_2_O. The research result is consistent with previous experimental results [24], which found that the formation of NH_3_ would be promoted by increasing the concentration of H_2_O in the feeding gas. Moreover, Tian et al. [28] proposed that the high NH_3_ yield from char-N (pyrrolic-nitrogen) gasification in steam resulted from the increased availability of H radical derived from the steam. According to our calculations, it is the initial interaction of H_2_O and pyrrole that determines the final formation of NH_3_, especially based on the adj-OH-C_2_-H and adj-OH-N-H interactions, since H_2_O will attack the C_2_ position of pyrrole with its hydroxyl group in both interactions.

The mechanisms discussed in this paper only indicate the thermal decomposition of pyrrole to form HCN and NH_3_. However, according to the previous study [29], N-containing compounds might also undergo polymerization to form solid residues. Therefore, the effects of both solid residues and H_2_O during pyrrole pyrolysis are worthy of investigation in a future work.

## 4. Materials and Methods

### 4.1. Materials

**Figure 8:**
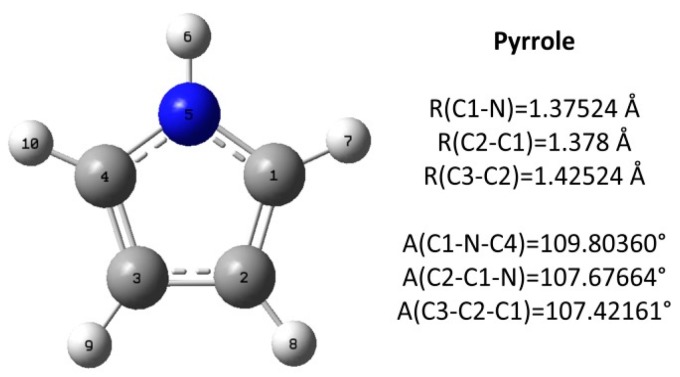
The molecular structure of pyrrole.

Pyrrole is a heterocyclic organic compound containing a five-membered ring with the formula C_4_H_4_NH. The optimized molecular structure and main parameters (bond length, bond angle) of pyrrole are shown in Figure 8. All carbon atoms are marked with numbers for convenient expression. Due to the symmetrical structure, there is no difference between the C_1_ group and C_4_ group, or between the C_2_ group and C_3_ group.

### 4.2. Methods

All calculations were carried out using the Gaussian 09 program [30]. The equilibrium geometries of the reactants, intermediates, transition states, and products were optimized by employing B3LYP/6-31G(d,p). DFT is a computational method that derives the properties of a system based on the electron density of the system [31,32]. The B3LYP functional with a 6-31G(d,p) basis set has been proven to be an appropriate choice for modeling organic species [31,33]. Moreover, DFT calculations have been successfully applied to investigate the pyrolysis mechanism of pyrrole model compounds [12,17].

In this work, the optimized structures of the reactants, intermediates, transition states, and products were further evaluated by frequency analysis, adopting the same method and basis set as the structural optimization calculations. The results of a frequency analysis were used to verify the stationary points to be minimal or first-order saddle points and to obtain their thermodynamic parameters. Intrinsic reaction coordinate (IRC) calculations were further performed to ensure the corresponding minimal and first-order saddle point on the same potential energy surface. Enthalpies were used for the discussion on energetics, under the standard conditions of 298.15 K and 1 atm. To evaluate the reliability of the selected calculation method and parameters, the structural parameters of a radical reaction were calculated and confirmed by triple spin multiplicity [31]. The energy barrier was calculated by the difference between the activation energy of reactants and concerted transition states (TS) [34].

## 5. Conclusions

In this study, the formation mechanisms of HCN and NH_3_ during pyrrole pyrolysis in the presence of H_2_O are investigated by DFT calculation. The results show that H_2_O and pyrrole can interact in four ways, followed by 13 possible pyrolysis pathways. Six pathways will result in the formation of NH_3_ (a-1, a-2, a-3, b-1, b-2, b-3), and another six pathways will lead to the HCN (a-4, a-5, c-2, c-3, c-4, d-1), while pathway c-1 does not lead to the formation of either HCN or NH_3_. Among all these pathways, pathways a-3 and b-1 are the most favorable ones, with an overall energy barrier of 362.3 and 361.4 kJ/mol, and both pathways result in the formation of NH_3_.

The formation of NH_3_ or HCN is determined by the different initial interactions of H_2_O with pyrrole. When H_2_O attacks the C_2_ position of pyrrole, there are two possible interactions (adj-OH-C_2_-H and adj-OH-N-H). In the following pathways based on the two interactions, NH_3_ will be formed while the formation of HCN is inhibited. When H_2_O attacks the C_3_ position of pyrrole with its hydroxyl group, it leads to another two interactions (ind-OH-C_1_-H and ind-OH-C_3_-H). In the following pathways, HCN will be generated instead of NH_3_. Meanwhile, the formation of HCN is inhibited compared to in reactions without H_2_O.

## Figures and Tables

**Table 1:** Comparison of NH_3_ and HCN formation from pyrrole in the presence of H_2_O.

Initial Interaction Way	Optimal Pathway	Overall Energy Barrier (kJ/mol)	Products
adj-OH-C_2_-H	a-3	362.3	NH_3_
adj-OH-N-H	b-1	361.4	NH_3_
ind-OH-C_1_-H	c-4	455.4	HCN
ind-OH-C_3_-H	d-1	413.9	HCN
